# Preventing Phrenic Nerve Stimulation by a Patch Insulation in an Intact Swine Heart Model

**DOI:** 10.1371/journal.pone.0102608

**Published:** 2014-07-17

**Authors:** Jin-Long Huang, Yenn-Jiang Lin, Yi-Wen Hung, Yu-Cheng Hsieh, Chien-Ming Cheng, Kuo-Yang Wang

**Affiliations:** 1 Cardiovascular Center, Taichung Veterans General Hospital, Taichung, Taiwan; 2 Institute of Clinical Medicine, and Cardiovascular Research Institute, Department of Medicine, School of Medicine, National Yang-Ming University, Taipei, Taiwan; 3 Department of Medicine, School of Medicine, Chung-Shan Medical University, Taichung, Taiwan; 4 Division of Cardiology, Department of Medicine, Taipei Veterans General Hospital, Taipei, Taiwan; 5 Department of Education and Research, Taichung Veterans General Hospital, Department of Veterinary Medicine, College of Veterinary Medicine, National Chung Hsing University, Taichung, Taiwan; 6 Division of Cardiology, Department of Medicine, Feng Yuan Hospital of the Ministry of Health and Welfare, Executive Yuan, Taichung City, Taiwan (R.O.C); University of Minnesota, United States of America

## Abstract

**Introduction:**

Phrenic nerve stimulation (PNS) could be prevented by a silastic patch over the epicardial lead. We studied the effects in preventing PNS by placing a silastic patch directly over an epicardial lead or placing a graft around the phrenic nerve (PN).

**Methods and Results:**

Fourteen Lanyu swine were enrolled. A bipolar lead was placed epicardially on the left ventricle (LV) inferior to the PN. An implantable cardioverter-defibrillator (ICD) lead was placed into the right ventricle (RV). The maximal influential distance (MID) was measured under 3 pacing configurations to express the influential electrical field on the PN. The threshold of the LV and PN were evaluated epicardially. Then, PTFE patches of different sizes (10×10 mm, 20×20 mm and 30×30 mm) were placed between the LV lead and PN to study the rise in PN threshold in 7 swine. On the other hand, the PN were surrounded by a PTFE graft of different lengths (10 mm, 20 mm, and 30 mm) in the remaining 7 swine. LV-bipolar pacing showed the shortest MID when compared to the other 2 unipolar pacing configurations at pacing voltage of 10 V. The patch was most effective in preventing PNS during LV-bipolar pacing. PNS was prevented under all circumstances with a larger PTFE patch (30×30 mm) or long graft (30 mm).

**Conclusions:**

PNS was avoided by placing a PTFE patch over the LV lead or a graft around the PN despite pacing configurations. Hence if PNS persisted during CRT implantation, a PTFE patch on the LV lead or a graft around the PN could be considered.

## Introduction

Cardiac resynchronization therapy (CRT) is an important therapeutic option in patients with heart failure who have mild to severe systolic left ventricular dysfunction with evidence of electric/mechanical dyssynchrony [1], [2]. Unintended stimulation of the phrenic nerve (PN) often occurs when the left ventricle (LV) is paced from the LV lead located in a preferred cardiac vein. This type of phrenic nerve stimulation (PNS) is found in over 37% of patients receiving CRT [3]. There are many ways to prevent PNS, including lead choices and adjustment of the pacing parameters. A newly-introduced quadripolar lead offers ten different LV pacing vectors to prevent PNS and improve the capture threshold at implant, thus obviating the need for lead repositioning post operation [4], [5]. However in some cases, electronic repositioning offered by a quadripolar LV lead was not able to overcome this problem and PNS still occurred as a result [6]. In this situation, re-implantation of LV lead or surgical approach was performed to prevent PNS by either a PTFE Gore-Tex patch placed directly over the LV lead or around the PN to shield the electrical field on the PN [7]–[9]. The electric field was defined as a function of the pacing energy (V/m), which dropped as the distance increased [10]. The maximal influential distance (MID) was measured as the maximal distance between the PN and LV lead tip that could cause PNS at a fixed pacing voltage and pulse width (0.5 ms) and was used in this study to express the effect of electrical field on the PN. This study will compare the effects of the 3 currently available LV pacing configurations (LV-tip to RV-coil, LV-tip to RV-ring and LV- bipolar) on the MID and PNS threshold in an open chest swine model. This model had been utilized in a study on PNS previously [11]. Furthermore, we examined the preferred size of the PTFE Gore-Tex patch required over the LV lead or around PN to prevent PNS.

## Methods

### Surgical preparation

**Figure 1:**
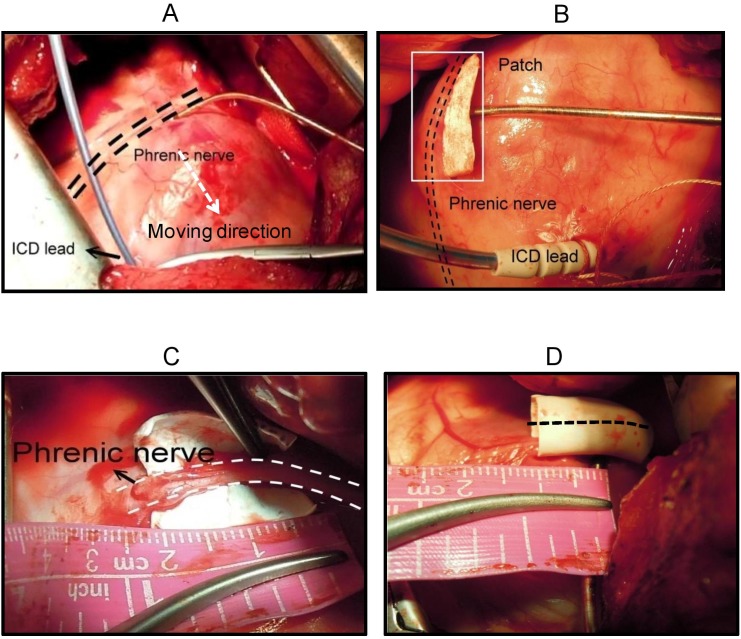
Panel A: A commercially available 1.1 mm tip-to-ring spaced LV pacing lead was inserted through a small hole created in the pericardium. The tip electrode position was maintained along the course of the left PN as the cathode, and the position was maintained inferior to the PN in order to keep homogeneous pacing conditions. An ICD lead with a single coil was then inserted into the RV using its ring or coil as the anode. The LV lead position was adjusted to measure the MID. Panel B: A PTEF patch was placed between the LV lead and the PN. Panel C: The PN was isolated from the pericardium and then a PTFE graft was placed around the PN. The black dotted line indicates the suture line. (Panel D).

Fourteen adult domestic Lanyu swine (30 to 35 kgs) were used in the study. All procedures were approved by the Animal Care Committee of the Taichung General Hospital and were performed according to the Guide to the Care and Use of Experimental Animals on Animal Care and the Guide for the Care and Use of Laboratory Animals published by the National Institutes of Health in Taiwan. All surgical thoracotomy procedures were performed while the swine were under general anesthesia. The animals were pre-medicated with ketamine (20 to 25 mg/kg, intramuscularly) and sodium pentobarbital (30 mg/kg IV). After intubation, anesthesia was maintained with 4% to 5% isoflurane and mechanically ventilated with room air. After performing a left thoracotomy, the hearts were exposed. A LV lead (1.1 mm distance between the tip and ring electrodes, OptiSense 1999, St. Jude Medical, Sylmar, CA, USA) was inserted into the pericardial sac and placed on the epicardium right under the PN by visual confirmation to keep homogeneous pacing conditions [11] (Figure 1). Then, an implantable cardioverter-defibrillator (ICD) lead with a single coil (Durata, 7122, St. Jude Medical Inc., Sylmar, CA, USA) was inserted into the right ventricular (RV) apex via the pulmonary trunk. The ring and coil of the ICD lead served as the anode during unipolar LV pacing. The surface electrocardiograms were monitored continuously on the programmer (Model 3510, St. Jude Medical, USA) throughout the entire study. The animals were housed and monitored in the swine lab by the veterinary (Hung YW). After the experiment, these animals were performed euthanasia by intravenous KCl (1–2 meq/kg) during anesthesia.

### Maximal influential distance and threshold measurements of the Myocardium and PN

Previous studies suggested that the occurrence of PNS depends on the proximity of the PN to the LV pacing cathode and the strength of the electric field [12]. The electric field was defined as a function of the pacing energy (V/m), which dropped as the distance increased [10]. The maximal influential distance (MID) was measured as the maximum distance between the PN and LV lead tip that would cause PNS under the different pacing voltages (1–10 V) with a 0.5 ms pulse width (PW). The MID (mm) was defined as the longest activation distance to the PN by the electrical field during local pacing. The MIDs were measured under 3 pacing configurations: LV unipolar pacing with LV tip as the cathode and RV coil as the anode (LV-tip to RV-coil), LV unipolar pacing with LV tip as the cathode and RV ring as the anode (LV-tip to RV-coil) and LV bipolar pacing.

After the measurement of the MID, the LV lead was fixed onto the epicardium inferior to the PN. The thresholds of the LV and PN were then determined using the 3 pacing configurations mentioned above. The stimulation amplitude started at 10 V and decreased at 0.1 volt steps. The LV threshold was defined as the lowest voltage that caused LV capture. The PN threshold was defined as the lowest voltage that induced diaphragmatic contractions [11]. Threshold measurements were repeated twice for each condition, and the average threshold of the two was adopted as the final threshold. Subsequently, the LV was paced at 180 beats/min to measure the LV threshold at various pulse durations (0.1–2.5 ms) [11]. In 7 out of 14 swine, the isolated patch (PTFE Gore-Tex patch, Bard Cardiovascular Patch 007837, Bard Inc., AZ 85281, USA) was placed over the LV electrode where the LV lead tip is located in the center of the patch (Figure 1, panel B). Three different patch sizes were tested: 10×10 mm; 20×20 mm; and 30×30 mm. In the remaining 7 swine, the PNs were insulated with the PTFE graft (Gore-Tex Stretch Vascular Graft W. L. Gore & Associates, Inc. Flagstaff, Arizona 86004, USA) at 3 different lengths (10 mm, 20 mm, and 30 mm) where the LV lead tip is located perpendicularly to the middle of the graft. The PN threshold was measured under different PWs (0.5, 1.0, 1.5, 2.0, 2.5 ms).

### Statistics

All parametric data were expressed as the mean±SD. A paired Student *t* test was used for paired comparisons. A one way ANOVA with a Turkey’s post hoc analysis was used for the comparisons among the three different pacing configurations. Mann-Whitney rank sum tests for pair comparisons and an ANOVA on ranks for comparisons of multiple groups were used to compare the non-parametric data. The correlation between pulse width and threshold of myocardium or PN used the Pearson correlation test. A *p* value of <0.05 was considered statistically significant. See File S1 for study data if necessary.

## Results

### Maximal Influential Distance (MID)

**Figure 2:**
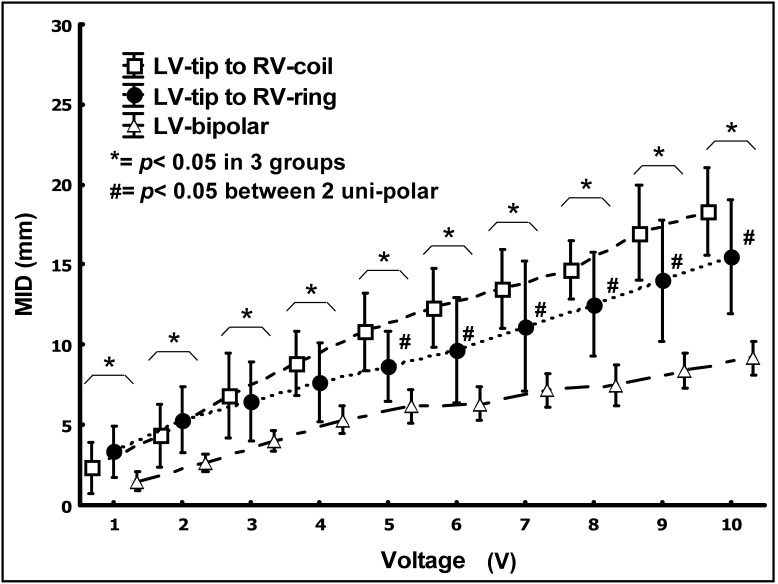
The MID was shortest during LV-bipolar pacing. Unipolar pacing configuration by the LV-tip to RV-ring had the shorter MID than the LV-tip to RV-coil pacing configuration when the pacing voltage over 5 V. * = *p*<0.05 indicated comparisons among the 3 groups (LV-tip to RV-coil vs. LV-tip to RV-ring vs. LV-bipolar), **#** = *p*<0.05 indicated comparisons between the 2 unipolar pacings (LV-tip to RV-coil vs. LV-tip to RV-ring).

Figure 2 shows the MIDs (the distance between the LV cathode and PN) with PNS at a PW of 0.5 ms under the 3 pacing configurations. LV-bipolar pacing had the shortest MID among the 3 pacing configurations from 1 V (1.5±0.5, 2.5±1.5 and 3.3±1.3 mm for LV-bipolar, LV-tip to RV-ring and LV-tip to RV coil respectively with *p*<0.05) to 10 V (8.3±0.8, 15.5±3.2, and 18.3±2.7 mm respectively with *p*<0.05). When the voltage was adjusted to the maximum value available in clinical practice at 8 V, LV-bipolar pacing again had the shortest MID (7.5±1.1, 12.4±1.6, and 14.8±1.1 mm for LV-bipolar, LV-tip to RV-ring and LV-tip to RV coil respectively with p<0.05). Between the two unipolar pacing configurations, LV-tip to RV-ring had the shorter MID compared with LV-tip to RV-coil when the pacing voltage was above 5 V. Although there was a difference in MID above 5 V, anodal stimulation at the RV occurred when the pacing voltage was abov 4 V in the LV-tip to RV-ring pacing. Mostly, the MID were less than 20 mm in all 3 pacing configurations at pacing voltages up to 10 V.

### The Strength–Duration Curve of Myocardium and PN

**Figure 3:**
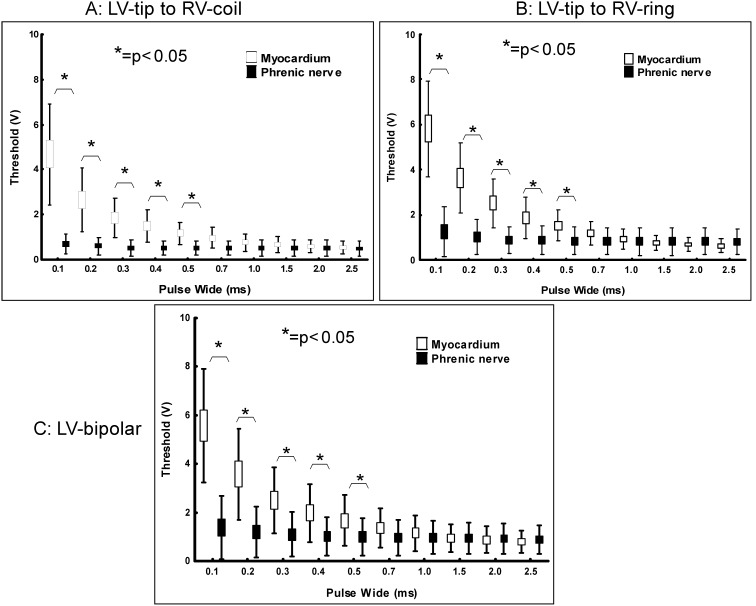
The strength–duration curves with the LV-tip to RV-coil (panel A), LV-tip to RV-ring (panel B), and LV-bipolar (panel C) pacing configurations. The strength–duration curve of the LV had a steeper slope than that of the PN. * = *p*<0.05 indicated comparisons between the thresholds of myocardium and PN in the same pulse width.

With the 3 pacing configurations, the threshold of the PN was significantly lower than that of the LV at PWs between 0.1 and 0.5 ms. The thresholds for the LV decreased as PW increased (r = –0.54, p<0.05). The strength-duration curve showed that LV had a steeper slope than PN (Figure 3A, 3B, 3C). In the LV-tip to RV-ring pacing configuration (Figure 3B), the LV threshold seemed lower than the PN threshold at PWs of 2.0 ms but statistical difference was not reached (0.6±0.3 and 0.8±0.6 V for LV and PN respectively with p = 0.54) and 2.5 ms (0.6±0.3 and 0.8±0.6 V for LV and PN respectively with p = 0.33)., The PN threshold was similar among all three pacing configurations.

### The PNS Prevention of Patches on the LV Leads

**Figure 4:**
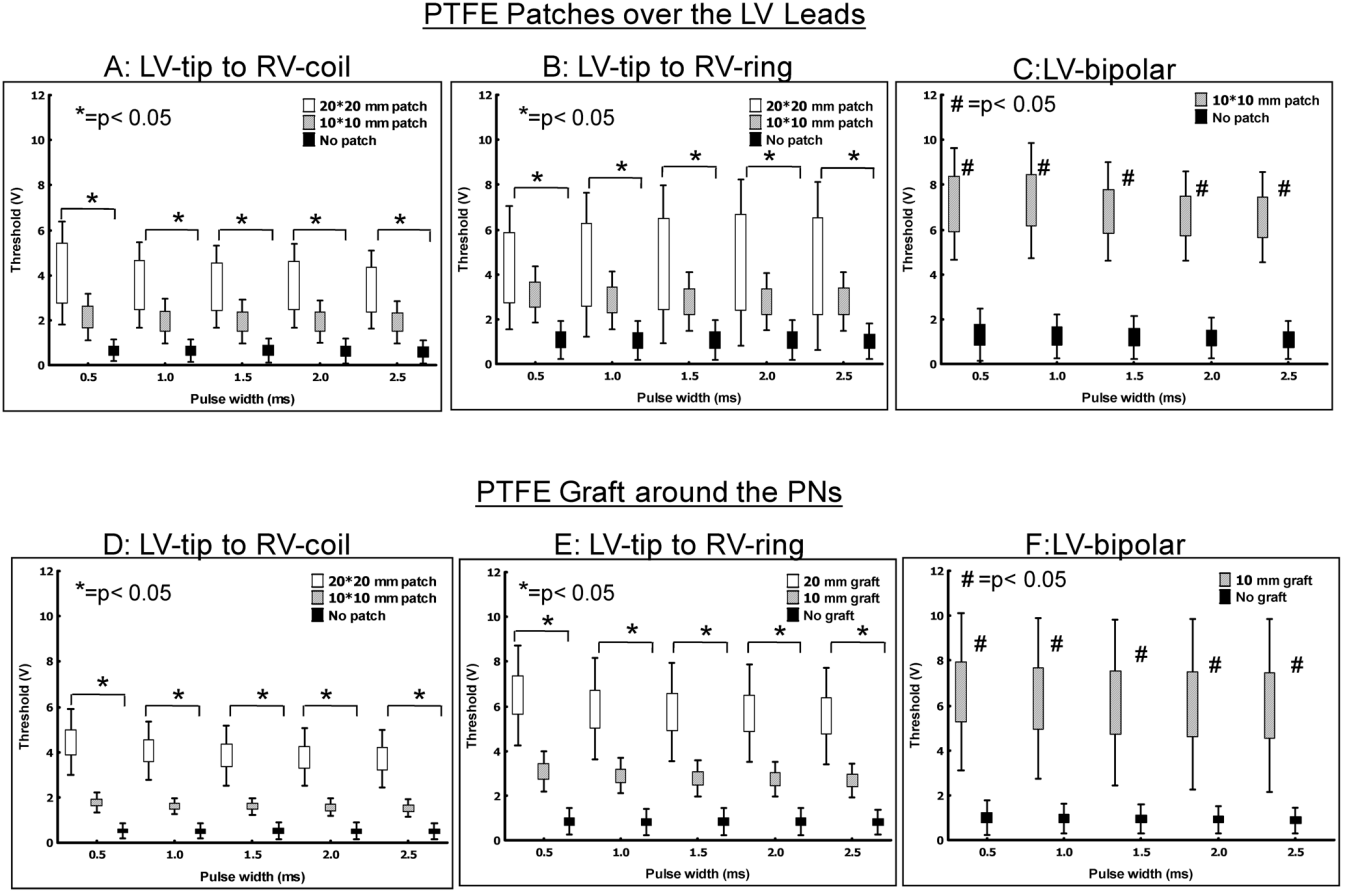
The changes in PN threshold after the patch was placed between the LV lead and PN during LV-tip to RV- coil pacing (panel A), LV-tip to RV-ring pacing (panel B) and LV-bipolar pacing (Panel C). Insulation of the PN by surrounding it with a layer of PTFE graft showed an increase in PN threshold among all three pacing configurations (Panel D, E, and F). * = *p*<0.05 comparisons of PN thresholds in the unipolar pacing (No patch vs.10×10 mm patch vs.20×20 mm patch in panel A and B; No graft vs.10 mm graft vs. 20 mm graft in panel C and D). # = *p*<0.05 comparisons of PN thresholds in the LV-bipolar pacing (No patch vs.10×10 mm patch in panel C; no graft vs. 10 mm graft in panel F).

The patches had significant effects on the increase in PN threshold among the 3 pacing configurations. The PN threshold increased more significantly with the application of the larger patch (20×20 mm) than with the smaller patch (10×10 mm). The PN threshold increased in the different PWs after the placement of patches (Figure 4). In the LV-tip to RV-coil configuration, the PN threshold increased from 0.4±0.1 V (no patch) to 2.1±0.7 V (10×10 mm patch) and 4.2±0.6 V (20×20 mm patch) (p<0.05). With the LV-tip to RV-ring configuration, the PN threshold also increased from 0.7±0.2 V (no patch) to 3.0±1.0 V (10×10 mm patch) and 4.4±1.0 V (20×20 mm patch) (p<0.05). With the LV-bipolar pacing configuration, the PN threshold increased from 0.7±0.2 V (no patch) to 6.5±1.5 V (10×10 mm patch) (p<0.05), while no PNS was observed at a pacing voltage 10 V with the 20×20 mm patch. When the patch size was 30 mm, no PNS was found in all these three pacing configurations. When comparing the insulation effect of the patch among the 3 pacing configurations, the PN threshold had the greatest increase in the LV-bipolar pacing configuration, followed by the LV-tip to RV-ring configuration. The LV-tip to RV-coil had the least increase in PN threshold.

### Insulation of PN by PTFE Graft

PNS could be avoided by surrounding the PN with a layer of PTFE graft in the other 7 swine to insulate the PN from the LV lead. The PN threshold increased progressively across all PWs (Figure 4D, E, F). For the LV-tip to RV-coil configuration, the PN threshold increased from 0.6±0.4 V (no graft) to 1.6±0.4 V (10 mm graft) and 4.0±1.3 V (20 mm graft) (p<0.05). With the LV-tip to RV-ring configuration, the PN threshold also increased from 1.1±0.8 V (no graft) to 2.8±0.8 V (10 mm graft) and 5.9±2.1 V (20 mm graft) (p<0.05). With the LV-bipolar pacing configuration, the PN threshold increased from 1.1±0.8 V (no graft) to 6.0±3.1 V (10 mm graft) (p<0.05), while no PNS was observed at a pacing voltage 10 V with the 20 mm graft. When the PTFE graft size was 30 mm, no PNS was found in all three pacing configurations. When comparing the insulation effect of the patch among the 3 pacing configurations, the PN threshold had the greatest increase in the LV-bipolar pacing, followed by the LV-tip to RV-ring configuration. At the end of the study, the PNS threshold was reexamined to ensure that there was no significant injury to the PN. Fortunately, the PNS thresholds in the LV-bipolar pacing did not change significantly after the procedures (baseline vs post procedure of PN threshold = 1.1±0.8 V vs 1.2±0.9 V, p = 0.08).

## Discussion

### Main findings

The main findings in this study were: (1) the MIDs were almost less than 20 mm in all 3 pacing configurations; (2) the LV-bipolar pacing configuration using a narrow inter-electrode spaced LV lead had the smallest MID among the 3 pacing configurations, suggesting that LV-bipolar pacing had the smallest electrical field; (3) PNS could be prevented by either placing a PTEF patch on the LV lead or a graft around the PN.

### Maximal Influential Distance and the Electrical Field

Iatrogenic PNS is a well-known consequence of LV pacing. PNS may cause the diaphragm to undesirably contract. PNS may feel like hiccups to the patient. PNS can occur when the LV pacing lead is close to the PN and is at a stimulation output that is strong enough to capture the nerve [13]. From experimental observations in canines, it was found that the stimulus was transmitted to the PN independent of pericardial nervous connections. The stimulus was mediated only through the PN by the spread of the electrical impulse from the epicardium to the overlying PN [13]. In fact, PNS was found to be dependent on the proximity to the LV pacing cathode and on the strength of the electric field applied in a recent study [12]. The electric field was expressed as a function of the pacing energy (V/m) and decreased with the increase in distance [10]. In this study, the MID was used to evaluate the influential distance of the electric field on the PN. The LV-bipolar pacing configuration using a LV lead with narrow inter-electrode spacing resulted in the shortest MID, followed by the LV-tip to RV-ring unipolar pacing configuration. The longest MID was observed during the LV-tip to RV-coil unipolar pacing configuration, indicating that this pacing configuration resulted in a wider electrical field. Most PN threshold were above 10 V if the distance between the LV lead and PN was over 20 mm, which was consistent with the study by Biffi et al [14]. Biffi et al. also showed that the PN threshold rapidly increased when the cathode moved away from the PN, reaching a PN threshold of 10 V at a 0.5 ms PW with a distance of 20 mm. In the conventional bipolar LV leads, the inter-electrode spaces were between 20 and 21 mm (QuickFlex, EasyTrak, Attain leads). Therefore, electronic repositioning might solve most PNS with multipolar leads [15]. Besides, in clinical unipolar pacing conditions, it is an option to select a pacing configuration with an LV-tip to RV-ring configuration which may also prevent PNS if PNS is intractable with the LV-tip to RV-coil pacing configuration. However, anodal stimulation would be a problem in this situation [16], [17] if the pacing voltage was above 4 V. This is due to a higher electrical density at the anodal site [18], hence the anode is usually designed as an electrode with a larger surface area to reduce the chance of anodal stimulation [16], [18]. Therefore, the LV-tip to RV-coil was chosen to avoid the anodal stimulation. But the electrical field would increase in our study by the longest MID.

The LV-bipolar pacing configuration using a narrow inter-electrode spaced LV lead has the shortest MID among the 3 pacing configurations, suggesting that LV-bipolar pacing would have the smallest electrical field among these 3 pacing configurations. LV-bipolar pacing with narrow inter-electrode spacing would be the ideal pacing configuration for pacing the LV without PNS.

### The Strength–Duration Curve

A previous study showed that the strength–duration relationship of the PN differs from that of the LV. LV pacing without PNS could be achieved by pacing with a long PW especially during unipolar pacing [11]. In this study, all 3 pacing configurations had a trend toward a lower threshold for the LV than that of the PN at a PW≥2 ms but did not reach statistical significance (Figure 3). Besides, it is not clinically practical to increase the PW because many pacemakers do not have the options for increasing PW up to 2.0 ms. Furthermore, the difference between the LV and PN threshold by the long PWs was very small (<1 V) [11]. Therefore, to prevent PNS, it would be practical to use a narrow inter-electrode spaced LV bipolar lead, electronic repositioning, or via insulation with patches.

### Effects of Patches on the PNS

PNS is a very important clinical issue because it occurs frequently at the same pacing sites where reverse remodeling occurs after CRT delivery [3]. To prevent PNS with the epicardial LV lead, a patch could be sown over the LV pacing electrode to shield it from the PN [7]. Van Steenberghe et al. reported that surgical insulation of the PN via a left anterolateral thoracotomy successfully abolished PNS at 6-month follow-up [9]. They used a Gore-Tex patch like the patches used in our study. With the thoracoscopic approach, a smaller patch would be suitable. Therefore, this study showed that a salvage patch can shield the PN from being captured by the LV lead. The greatest effect could be achieved by LV-bipolar pacing because it had the smallest MID and could be easily shielded by a small patch. Insulation of PN by surrounding it with a layer of the PTFE graft was an alternative method in preventing PNS. The insulation effect was similar to the PTFE patch over the LV leads. The PNS could be prevented if the PTFE patch or graft was over 30 mm. In the clinical implication, a larger PTFE patch (30×30 mm) would be needed to place between PN and LV lead to avoid the PNS if the electrical reposition could not solve the PNS. It is possible to have the risk of PN damage after the surgery to surrounding the PN with PTFE graft. Therefore, PN thresholds were measured before the after the procedure and showed no significant changes by the LV-bipolar pacing (baseline vs post procedure of PN threshold = 1.1±0.8 V vs. 1.2±0.9 V, p = 0.08).

### Study Limitations

This was an open chest model with the LV lead placed epicardially, which is different from placing the LV lead via the coronary sinus in the clinical transvenous approach. It may be easier to stimulate the PN epicardially than from the cardiac vein because of the close proximity to the PN. However, this model is similar to the surgical epicardial approach. This model is appropriate for evaluating the clinical situation via the surgical epicardial approach.

## Conclusions

During CRT implantation procedures, LV-bipolar pacing configuration with a narrow inter-electrode spaced LV lead is preferred for effective pacing the LV without PNS. If PNS persisted, a PTFE patch can be placed over the LV lead or a graft around the PN in order to prevent PNS.

## Supporting Information

File S1 **Measurement of MID (Maximal influential distance).**
(PDF)
