# Supplementary material for: Preventing Phrenic Nerve Stimulation by a Patch Insulation in an Intact Swine Heart Model
Source: PLoS One. 2014 Jul 17;9(7):e102608. doi: 10.1371/journal.pone.0102608 (PMC4102517; doi:10.1371/journal.pone.0102608)
Supplement: File S1 — Measurement of MID (Maximal influential distance). (PDF) [file pone.0102608.s001.pdf]

Mesurement of MID (Maximal influential distance) (mm)

	Pacing Voltage (V)	LV-tip to RV-coil(mm)	LV-tip to RV-ring(mm)	LV-bi-polar(mm)
pig1	1	5	6	2
pig2	1	5	5	2
pig3	1	5	2	1
pig4	1	5	2	2
pig5	1	3	4	2
pig6	1	2	5	1
pig7	1	4	1	2
pig8	1	2	2	1
pig9	1	2	1	1
pig10	1	2	1	2
pig11	1	2	1	1
pig12	1	3	1	1
pig13	1	4	3	2
pig14	1	2	1	1
pig1	2	5	8	3
pig2	2	7	6	3
pig3	2	7	6	3
pig4	2	6	6	3
pig5	2	2	3	2
pig6	2	3	3	2
pig7	2	3	2	2
pig8	2	3	3	1
pig9	2	4	2	1
pig10	2	5	3	3
pig11	2	5	4	3
pig12	2	5	3	2
pig13	2	6	4	4
pig14	2	3	2	1
pig1	3	8	10	4
pig2	3	11	9	4
pig3	3	7	6	4
pig4	3	8	4	5
pig5	3	5	5	3
pig6	3	4	4	4

pig7	3	4	4	3
pig8	3	5	4	2
pig9	3	6	3	2
pig10	3	6	6	4
pig11	3	6	6	4
pig12	3	6	6	3
pig13	3	7	6	6
pig14	3	6	4	2
pig1	4	9	10	5
pig2	4	12	9	6
pig3	4	8	7	6
pig4	4	9	10	6
pig5	4	9	7	4
pig6	4	6	7	5
pig7	4	5	6	4
pig8	4	7	7	4
pig9	4	7	4	2
pig10	4	7	7	5
pig11	4	6	5	4
pig12	4	9	5	4
pig13	4	8	7	7
pig14	4	7	5	3
pig1	5	13	11	6
pig2	5	13	9	6
pig3	5	12	8	6
pig4	5	10	11	8
pig5	5	10	7	5
pig6	5	7	6	6
pig7	5	6	4	5
pig8	5	8	7	5
pig9	5	8	5	4
pig10	5	9	6	5
pig11	5	6	6	5
pig12	5	11	7	5
pig13	5	9	8	7
pig14	5	9	6	5

pig1	6	15	13	5
pig2	6	15	9	7
pig3	6	12	8	6
pig4	6	14	14	8
pig5	6	11	8	6
pig6	6	9	6	6
pig7	6	8	6	6
pig8	6	9	8	6
pig9	6	10	6	4
pig10	6	10	7	6
pig11	6	7	6	6
pig12	6	12	8	5
pig13	6	11	9	8
pig14	6	10	7	5
pig1	7	16	15	6
pig2	7	16	10	8
pig3	7	14	9	8
pig4	7	15	15	8
pig5	7	12	8	6
pig6	7	10	8	7
pig7	7	9	7	6
pig8	7	12	11	6
pig9	7	12	8	4
pig10	7	10	7	7
pig11	7	8	7	7
pig12	7	14	9	6
pig13	7	12	9	8
pig14	7	10	8	6
pig1	8	16	15	6
pig2	8	16	11	9
pig3	8	16	12	8
pig4	8	15	13	8
pig5	8	16	13	6
pig6	8	15	14	8
pig7	8	13	12	8

pig8	8	16	15	6
pig9	8	14	13	6
pig10	8	15	11	8
pig11	8	14	9	7
pig12	8	13	12	8
pig13	8	14	12	9
pig14	8	14	12	8
pig1	9	19	17	7
pig2	9	18	12	10
pig3	9	16	15	8
pig4	9	21	15	8
pig5	9	18	13	7
pig6	9	16	13	9
pig7	9	14	10	9
pig8	9	17	16	7
pig9	9	15	11	7
pig10	9	16	12	7
pig11	9	15	15	7
pig12	9	15	13	9
pig13	9	16	14	8
pig14	9	15	13	9
pig1	10	22	20	8
pig2	10	17	16	9
pig3	10	18	21	9
pig4	10	22	15	9
pig5	10	20	13	8
pig6	10	16	14	9
pig7	10	15	10	7
pig8	10	21	17	7
pig9	10	15	11	8
pig10	10	16	15	8
pig11	10	17	15	8
pig12	10	16	14	8
pig13	10	21	17	10
pig14	10	20	19	8

Measurement of myocardial and phrenic nerve threshold by LV-tip to RV-coil pacing

		Myocardial threshold(MT)			PN threshold (PNT)		
	Pulse width (ms)	MT-1	MT-2	Mean	PNT-1	PNT-2	Mean
pig 1	0.1	1.8	1.7	1.75	0.4	0.4	0.4
pig 1	0.2	0.7	0.8	0.75	0.3	0.4	0.35
pig 1	0.3	0.6	0.6	0.6	0.4	0.4	0.4
pig 1	0.4	0.5	0.6	0.55	0.4	0.4	0.4
pig 1	0.5	0.4	0.4	0.4	0.4	0.4	0.4
pig 1	0.7	0.4	0.4	0.4	0.4	0.4	0.4
pig 1	1	0.3	0.3	0.3	0.4	0.4	0.4
pig 1	1.5	0.3	0.3	0.3	0.4	0.4	0.4
pig 1	2	0.2	0.2	0.2	0.4	0.4	0.4
pig 1	2.5	0.2	0.2	0.2	0.4	0.4	0.4
pig 2	0.1	5.3	5.4	5.35	0.6	0.7	0.65
pig 2	0.2	2.7	2.8	2.75	0.6	0.6	0.6
pig 2	0.3	2.1	2	2.05	0.5	0.5	0.5
pig 2	0.4	1.7	1.7	1.7	0.5	0.5	0.5
pig 2	0.5	1.5	1.6	1.55	0.5	0.5	0.5
pig 2	0.7	1.3	1.3	1.3	0.5	0.5	0.5
pig 2	1	1.1	1.1	1.1	0.5	0.5	0.5
pig 2	1.5	1	1	1	0.5	0.5	0.5
pig 2	2	0.9	1	0.95	0.5	0.5	0.5
pig 2	2.5	0.9	0.9	0.9	0.5	0.5	0.5
pig 3	0.1	7	7.1	7.05	0.5	0.5	0.5
pig 3	0.2	3.1	3.1	3.1	0.4	0.4	0.4
pig 3	0.3	2.1	2.1	2.1	0.4	0.4	0.4
pig 3	0.4	1.4	1.5	1.45	0.4	0.4	0.4
pig 3	0.5	1.4	1.4	1.4	0.4	0.4	0.4
pig 3	0.7	1.2	1.2	1.2	0.4	0.4	0.4
pig 3	1	1.1	1.1	1.1	0.4	0.4	0.4
pig 3	1.5	1	1	1	0.4	0.4	0.4
pig 3	2	0.9	0.9	0.9	0.4	0.4	0.4
pig 3	2.5	0.8	0.8	0.8	0.4	0.4	0.4
pig 4	0.1	5.1	5	5.05	0.6	0.5	0.55

pig 4	0.2	4.1	4.1	**4.1**	0.3	0.3	**0.3**
pig 4	0.3	3.4	3.4	**3.4**	0.3	0.3	**0.3**
pig 4	0.4	2.6	2.8	**2.7**	0.3	0.3	**0.3**
pig 4	0.5	2	2	**2**	0.3	0.3	**0.3**
pig 4	0.7	1.3	1.3	**1.3**	0.3	0.3	**0.3**
pig 4	1	0.5	0.5	**0.5**	0.3	0.3	**0.3**
pig 4	1.5	0.4	0.4	**0.4**	0.3	0.3	**0.3**
pig 4	2	0.3	0.3	**0.3**	0.3	0.3	**0.3**
pig 4	2.5	0.2	0.2	**0.2**	0.3	0.3	**0.3**
pig 5	0.1	6	6.2	**6.1**	0.6	0.6	**0.6**
pig 5	0.2	4	4.1	**4.05**	0.6	0.6	**0.6**
pig 5	0.3	2.2	2.2	**2.2**	0.5	0.5	**0.5**
pig 5	0.4	1.8	1.8	**1.8**	0.5	0.5	**0.5**
pig 5	0.5	1.3	1.3	**1.3**	0.5	0.5	**0.5**
pig 5	0.7	1.1	1.2	**1.15**	0.5	0.5	**0.5**
pig 5	1	0.8	0.8	**0.8**	0.5	0.5	**0.5**
pig 5	1.5	0.8	0.8	**0.8**	0.5	0.5	**0.5**
pig 5	2	0.7	0.7	**0.7**	0.5	0.5	**0.5**
pig 5	2.5	0.7	0.7	**0.7**	0.5	0.5	**0.5**
pig 6	0.1	4.3	4.5	**4.4**	0.4	0.3	**0.35**
pig 6	0.2	2.4	2.4	**2.4**	0.2	0.2	**0.2**
pig 6	0.3	1.6	1.7	**1.65**	0.2	0.2	**0.2**
pig 6	0.4	1.2	1.2	**1.2**	0.2	0.2	**0.2**
pig 6	0.5	1	1.1	**1.05**	0.2	0.2	**0.2**
pig 6	0.7	0.8	0.8	**0.8**	0.2	0.2	**0.2**
pig 6	1	0.6	0.6	**0.6**	0.2	0.2	**0.2**
pig 6	1.5	0.5	0.5	**0.5**	0.2	0.2	**0.2**
pig 6	2	0.4	0.4	**0.4**	0.2	0.2	**0.2**
pig 6	2.5	0.4	0.4	**0.4**	0.2	0.2	**0.2**
pig 7	0.1	4.5	4.6	**4.55**	0.7	0.7	**0.7**
pig 7	0.2	3.2	3.2	**3.2**	0.6	0.6	**0.6**
pig 7	0.3	2.5	2.5	**2.5**	0.5	0.5	**0.5**
pig 7	0.4	2	2.1	**2.05**	0.5	0.5	**0.5**
pig 7	0.5	1.6	1.6	**1.6**	0.5	0.5	**0.5**
pig 7	0.7	1.3	1.3	**1.3**	0.5	0.5	**0.5**

pig 7	1	1	1	**1**	0.5	0.5	**0.5**
pig 7	1.5	0.7	0.7	**0.7**	0.5	0.5	**0.5**
pig 7	2	0.7	0.7	**0.7**	0.5	0.5	**0.5**
pig 7	2.5	0.6	0.6	**0.6**	0.5	0.5	**0.5**
pig 8	0.1	5.2	5	**5.1**	0.4	0.4	**0.4**
pig 8	0.2	2.1	2	**2.05**	0.3	0.3	**0.3**
pig 8	0.3	1.8	1.7	**1.75**	0.3	0.3	**0.3**
pig 8	0.4	1.5	1.6	**1.55**	0.3	0.3	**0.3**
pig 8	0.5	1.3	1.3	**1.3**	0.3	0.3	**0.3**
pig 8	0.7	0.9	0.9	**0.9**	0.3	0.3	**0.3**
pig 8	1	0.8	0.8	**0.8**	0.3	0.3	**0.3**
pig 8	1.5	0.7	0.7	**0.7**	0.3	0.3	**0.3**
pig 8	2	0.7	0.7	**0.7**	0.2	0.2	**0.2**
pig 8	2.5	0.7	0.7	**0.7**	0.2	0.2	**0.2**
pig 9	0.1	2.2	2.3	**2.25**	0.9	1	**0.95**
pig 9	0.2	1.3	1.2	**1.25**	0.7	0.7	**0.7**
pig 9	0.3	0.8	0.8	**0.8**	0.6	0.6	**0.6**
pig 9	0.4	0.7	0.7	**0.7**	0.6	0.6	**0.6**
pig 9	0.5	0.6	0.6	**0.6**	0.5	0.5	**0.5**
pig 9	0.7	0.4	0.4	**0.4**	0.5	0.5	**0.5**
pig 9	1	0.4	0.4	**0.4**	0.5	0.5	**0.5**
pig 9	1.5	0.3	0.3	**0.3**	0.5	0.5	**0.5**
pig 9	2	0.3	0.3	**0.3**	0.5	0.5	**0.5**
pig 9	2.5	0.3	0.3	**0.3**	0.5	0.5	**0.5**
pig 10	0.1	1.7	1.6	**1.65**	1.9	2	**1.95**
pig 10	0.2	0.9	0.9	**0.9**	1.7	1.7	**1.7**
pig 10	0.3	0.7	0.7	**0.7**	1.6	1.6	**1.6**
pig 10	0.4	0.5	0.6	**0.55**	1.5	1.5	**1.5**
pig 10	0.5	0.5	0.5	**0.5**	1.5	1.5	**1.5**
pig 10	0.7	0.4	0.4	**0.4**	1.5	1.5	**1.5**
pig 10	1	0.3	0.3	**0.3**	1.6	1.6	**1.6**
pig 10	1.5	0.3	0.3	**0.3**	1.7	1.7	**1.7**
pig 10	2	0.3	0.3	**0.3**	1.7	1.7	**1.7**
pig 10	2.5	0.3	0.3	**0.3**	1.6	1.6	**1.6**
pig 11	0.1	9.6	9.8	**9.7**	1.2	1.3	**1.25**

pig 11	0.2	5.5	5.7	**5.6**	1.2	1.2	**1.2**
pig 11	0.3	3.1	3.2	**3.15**	1	1	**1**
pig 11	0.4	2.7	2.8	**2.75**	0.9	0.9	**0.9**
pig 11	0.5	1.8	1.8	**1.8**	0.9	0.9	**0.9**
pig 11	0.7	2	2	**2**	0.9	0.9	**0.9**
pig 11	1	1.7	1.7	**1.7**	0.8	0.8	**0.8**
pig 11	1.5	1.6	1.6	**1.6**	0.7	0.7	**0.7**
pig 11	2	1.1	1.1	**1.1**	0.7	0.7	**0.7**
pig 11	2.5	1.1	1.1	**1.1**	0.6	0.6	**0.6**
pig 12	0.1	4	3.9	**3.95**	0.4	0.4	**0.4**
pig 12	0.2	2.1	2.1	**2.1**	0.4	0.4	**0.4**
pig 12	0.3	1.6	1.5	**1.55**	0.4	0.4	**0.4**
pig 12	0.4	1.3	1.3	**1.3**	0.4	0.4	**0.4**
pig 12	0.5	1	1.1	**1.05**	0.4	0.4	**0.4**
pig 12	0.7	0.8	0.8	**0.8**	0.4	0.4	**0.4**
pig 12	1	0.7	0.7	**0.7**	0.4	0.4	**0.4**
pig 12	1.5	0.6	0.6	**0.6**	0.4	0.4	**0.4**
pig 12	2	0.5	0.5	**0.5**	0.4	0.4	**0.4**
pig 12	2.5	0.4	0.4	**0.4**	0.4	0.4	**0.4**
pig 13	0.1	3.6	3.7	**3.65**	0.6	0.7	**0.65**
pig 13	0.2	1.9	2.1	**2**	0.6	0.6	**0.6**
pig 13	0.3	1.5	1.5	**1.5**	0.5	0.5	**0.5**
pig 13	0.4	1.2	1.1	**1.15**	0.5	0.5	**0.5**
pig 13	0.5	1	1	**1**	0.5	0.5	**0.5**
pig 13	0.7	0.8	0.8	**0.8**	0.5	0.5	**0.5**
pig 13	1	0.7	0.7	**0.7**	0.5	0.5	**0.5**
pig 13	1.5	0.6	0.6	**0.6**	0.5	0.5	**0.5**
pig 13	2	0.6	0.6	**0.6**	0.5	0.5	**0.5**
pig 13	2.5	0.6	0.6	**0.6**	0.5	0.5	**0.5**
pig 14	0.1	5	5.1	**5.05**	0.4	0.4	**0.4**
pig 14	0.2	2.1	2	**2.05**	0.3	0.4	**0.35**
pig 14	0.3	1.7	1.7	**1.7**	0.3	0.3	**0.3**
pig 14	0.4	1.5	1.4	**1.45**	0.3	0.3	**0.3**
pig 14	0.5	1.3	1.3	**1.3**	0.3	0.3	**0.3**
pig 14	0.7	0.9	0.9	**0.9**	0.3	0.3	**0.3**

pig 14	1	0.8	0.8	**0.8**	0.3	0.3	**0.3**
pig 14	1.5	0.7	0.7	**0.7**	0.3	0.3	**0.3**
pig 14	2	0.7	0.7	**0.7**	0.3	0.3	**0.3**
pig 14	2.5	0.7	0.7	**0.7**	0.3	0.3	**0.3**

Measurement of myocardial and phrenic nerve threshold by LV-tip to RV-ring pacing

LV-tip to RV-ring		Myocardial threshold(MT)			PN threshold (PN)		
	Pulse width (ms)	MT-1	MT-2	mean	PNT-1	PNT-2	mean
pig 1	0.1	3	3.1	**3.05**	0.6	0.6	**0.6**
pig 1	0.2	1.5	1.6	**1.55**	0.6	0.6	**0.6**
pig 1	0.3	1	1	**1**	0.6	0.6	**0.6**
pig 1	0.4	0.7	0.7	**0.7**	0.5	0.5	**0.5**
pig 1	0.5	0.6	0.6	**0.6**	0.5	0.5	**0.5**
pig 1	0.7	0.5	0.5	**0.5**	0.5	0.5	**0.5**
pig 1	1	0.4	0.4	**0.4**	0.5	0.5	**0.5**
pig 1	1.5	0.4	0.4	**0.4**	0.5	0.5	**0.5**
pig 1	2	0.3	0.3	**0.3**	0.5	0.5	**0.5**
pig 1	2.5	0.2	0.2	**0.2**	0.5	0.5	**0.5**
pig 2	0.1	6.7	6.8	**6.75**	1	1	**1**
pig 2	0.2	3.5	3.6	**3.55**	1	1	**1**
pig 2	0.3	2.6	2.6	**2.6**	0.8	0.9	**0.85**
pig 2	0.4	2.1	2.1	**2.1**	0.8	0.8	**0.8**
pig 2	0.5	1.9	1.8	**1.85**	0.8	0.8	**0.8**
pig 2	0.7	1.5	1.5	**1.5**	0.7	0.7	**0.7**
pig 2	1	1.3	1.3	**1.3**	0.7	0.7	**0.7**
pig 2	1.5	1.1	1.1	**1.1**	0.7	0.7	**0.7**
pig 2	2	1	1	**1**	0.8	0.8	**0.8**
pig 2	2.5	1	1	**1**	0.7	0.7	**0.7**
pig 3	0.1	7.6	7.4	**7.5**	0.8	0.8	**0.8**
pig 3	0.2	6.1	6.2	**6.15**	0.6	0.6	**0.6**
pig 3	0.3	4.2	4.3	**4.25**	0.6	0.6	**0.6**
pig 3	0.4	3.4	3.4	**3.4**	0.5	0.5	**0.5**
pig 3	0.5	2.2	2.2	**2.2**	0.5	0.5	**0.5**
pig 3	0.7	1.2	1.2	**1.2**	0.5	0.5	**0.5**
pig 3	1	1	1	**1**	0.5	0.5	**0.5**

pig 3	1.5	0.6	0.6	**0.6**	0.5	0.5	**0.5**
pig 3	2	0.5	0.5	**0.5**	0.5	0.5	**0.5**
pig 3	2.5	0.4	0.4	**0.4**	0.5	0.5	**0.5**
pig 4	0.1	5.2	5.4	**5.3**	1.2	1.3	**1.25**
pig 4	0.2	4.5	4.6	**4.55**	1	1	**1**
pig 4	0.3	2.7	2.8	**2.75**	1	1	**1**
pig 4	0.4	1	1	**1**	1	1	**1**
pig 4	0.5	0.9	0.9	**0.9**	1.1	1.1	**1.1**
pig 4	0.7	0.7	0.7	**0.7**	1	1	**1**
pig 4	1	0.4	0.4	**0.4**	1	1	**1**
pig 4	1.5	0.2	0.2	**0.2**	1	1	**1**
pig 4	2	0.2	0.2	**0.2**	1	1	**1**
pig 4	2.5	0.2	0.2	**0.2**	1	1	**1**
pig 5	0.1	6.6	6.9	**6.75**	0.8	0.9	**0.85**
pig 5	0.2	4.8	4.9	**4.85**	0.9	0.9	**0.9**
pig 5	0.3	2.9	3	**2.95**	0.8	0.8	**0.8**
pig 5	0.4	1.8	1.8	**1.8**	0.7	0.7	**0.7**
pig 5	0.5	1.6	1.5	**1.55**	0.7	0.7	**0.7**
pig 5	0.7	1.3	1.3	**1.3**	0.7	0.7	**0.7**
pig 5	1	1.1	1.1	**1.1**	0.7	0.7	**0.7**
pig 5	1.5	1	1	**1**	0.7	0.7	**0.7**
pig 5	2	0.9	0.9	**0.9**	0.7	0.7	**0.7**
pig 5	2.5	0.8	0.8	**0.8**	0.7	0.7	**0.7**
pig 6	0.1	8.3	8.5	**8.4**	0.4	0.4	**0.4**
pig 6	0.2	4.9	5	**4.95**	0.3	0.3	**0.3**
pig 6	0.3	3.2	3.3	**3.25**	0.3	0.3	**0.3**
pig 6	0.4	2.6	2.7	**2.65**	0.3	0.3	**0.3**
pig 6	0.5	2.2	2.2	**2.2**	0.3	0.3	**0.3**
pig 6	0.7	1.7	1.7	**1.7**	0.3	0.3	**0.3**
pig 6	1	1.1	1.1	**1.1**	0.3	0.3	**0.3**
pig 6	1.5	0.9	0.9	**0.9**	0.3	0.3	**0.3**
pig 6	2	0.8	0.8	**0.8**	0.3	0.3	**0.3**
pig 6	2.5	0.8	0.8	**0.8**	0.3	0.3	**0.3**
pig 7	0.1	6	6.1	**6.05**	1.2	1.1	**1.15**
pig 7	0.2	4.2	4.3	**4.25**	1.1	1.1	**1.1**

pig 7	0.3	2.9	2.9	**2.9**	1	1	**1**
pig 7	0.4	2.4	2.3	**2.35**	0.9	0.9	**0.9**
pig 7	0.5	1.9	1.9	**1.9**	0.8	0.8	**0.8**
pig 7	0.7	1.5	1.5	**1.5**	0.8	0.8	**0.8**
pig 7	1	1.1	1.1	**1.1**	0.8	0.8	**0.8**
pig 7	1.5	0.9	0.9	**0.9**	0.8	0.8	**0.8**
pig 7	2	0.8	0.8	**0.8**	0.8	0.8	**0.8**
pig 7	2.5	0.8	0.8	**0.8**	0.8	0.8	**0.8**
pig 8	0.1	2.1	2	**2.05**	0.7	0.6	**0.65**
pig 8	0.2	2	1.8	**1.9**	0.5	0.5	**0.5**
pig 8	0.3	1.6	1.7	**1.65**	0.4	0.4	**0.4**
pig 8	0.4	1.6	1.6	**1.6**	0.4	0.4	**0.4**
pig 8	0.5	1.3	1.4	**1.35**	0.4	0.4	**0.4**
pig 8	0.7	0.8	0.9	**0.85**	0.4	0.4	**0.4**
pig 8	1	0.8	0.8	**0.8**	0.4	0.4	**0.4**
pig 8	1.5	0.7	0.7	**0.7**	0.4	0.4	**0.4**
pig 8	2	0.7	0.7	**0.7**	0.4	0.4	**0.4**
pig 8	2.5	0.7	0.7	**0.7**	0.4	0.4	**0.4**
pig 9	0.1	3.2	3.3	**3.25**	1.2	1.3	**1.25**
pig 9	0.2	2.2	2.2	**2.2**	1	1	**1**
pig 9	0.3	1.4	1.3	**1.35**	0.9	0.9	**0.9**
pig 9	0.4	1	1	**1**	0.8	0.8	**0.8**
pig 9	0.5	0.8	0.8	**0.8**	0.8	0.8	**0.8**
pig 9	0.7	0.6	0.6	**0.6**	0.8	0.8	**0.8**
pig 9	1	0.5	0.5	**0.5**	0.7	0.7	**0.7**
pig 9	1.5	0.4	0.4	**0.4**	0.7	0.7	**0.7**
pig 9	2	0.4	0.4	**0.4**	0.7	0.7	**0.7**
pig 9	2.5	0.3	0.3	**0.3**	0.7	0.7	**0.7**
pig 10	0.1	3	3.1	**3.05**	4.3	4.5	**4.4**
pig 10	0.2	1.5	1.4	**1.45**	3.2	3.3	**3.25**
pig 10	0.3	1	1	**1**	2.6	2.6	**2.6**
pig 10	0.4	0.8	0.8	**0.8**	2.6	2.6	**2.6**
pig 10	0.5	0.8	0.8	**0.8**	2.5	2.5	**2.5**
pig 10	0.7	0.5	0.5	**0.5**	2.5	2.5	**2.5**
pig 10	1	0.5	0.5	**0.5**	2.6	2.6	**2.6**

pig 10	1.5	0.5	0.5	**0.5**	2.6	2.6	**2.6**
pig 10	2	0.5	0.5	**0.5**	2.6	2.6	**2.6**
pig 10	2.5	0.4	0.4	**0.4**	2.4	2.4	**2.4**
pig 11	0.1	9.8	10	**9.9**	2.6	2.8	**2.7**
pig 11	0.2	5.6	5.8	**5.7**	2.2	2.1	**2.15**
pig 11	0.3	4.3	4.3	**4.3**	1.6	1.6	**1.6**
pig 11	0.4	3.5	3.6	**3.55**	2	2	**2**
pig 11	0.5	2.9	2.9	**2.9**	1.7	1.7	**1.7**
pig 11	0.7	2.3	2.3	**2.3**	1.7	1.7	**1.7**
pig 11	1	2	2	**2**	1.5	1.5	**1.5**
pig 11	1.5	1.5	1.5	**1.5**	1.6	1.6	**1.6**
pig 11	2	1.4	1.4	**1.4**	1.6	1.6	**1.6**
pig 11	2.5	1.2	1.2	**1.2**	1.5	1.5	**1.5**
pig 12	0.1	4.6	4.5	**4.55**	0.6	0.6	**0.6**
pig 12	0.2	2.6	2.7	**2.65**	0.5	0.5	**0.5**
pig 12	0.3	2	2	**2**	0.4	0.4	**0.4**
pig 12	0.4	1.6	1.8	**1.7**	0.4	0.4	**0.4**
pig 12	0.5	1.5	1.5	**1.5**	0.4	0.4	**0.4**
pig 12	0.7	1.2	1.2	**1.2**	0.4	0.4	**0.4**
pig 12	1	0.9	0.9	**0.9**	0.4	0.4	**0.4**
pig 12	1.5	0.8	0.8	**0.8**	0.4	0.4	**0.4**
pig 12	2	0.7	0.7	**0.7**	0.4	0.4	**0.4**
pig 12	2.5	0.6	0.6	**0.6**	0.4	0.4	**0.4**
pig 13	0.1	5.3	5.2	**5.25**	1.1	1.1	**1.1**
pig 13	0.2	2.2	2.3	**2.25**	1	1	**1**
pig 13	0.3	1.7	1.8	**1.75**	0.9	0.9	**0.9**
pig 13	0.4	1.4	1.4	**1.4**	0.9	0.9	**0.9**
pig 13	0.5	1.1	1.1	**1.1**	0.9	0.9	**0.9**
pig 13	0.7	1	1	**1**	0.9	0.9	**0.9**
pig 13	1	0.8	0.8	**0.8**	0.9	0.9	**0.9**
pig 13	1.5	0.7	0.7	**0.7**	0.9	0.9	**0.9**
pig 13	2	0.6	0.6	**0.6**	0.9	0.9	**0.9**
pig 13	2.5	0.6	0.6	**0.6**	0.9	0.9	**0.9**
pig 14	0.1	5	4.9	**4.95**	0.4	0.5	**0.45**
pig 14	0.2	2.7	2.8	**2.75**	0.4	0.4	**0.4**

pig 14	0.3	2.4	2.4	**2.4**	0.4	0.4	**0.4**
pig 14	0.4	1.8	1.9	**1.85**	0.4	0.4	**0.4**
pig 14	0.5	1.5	1.5	**1.5**	0.4	0.4	**0.4**
pig 14	0.7	1.2	1.2	**1.2**	0.4	0.4	**0.4**
pig 14	1	1	1	**1**	0.4	0.4	**0.4**
pig 14	1.5	0.9	0.9	**0.9**	0.4	0.4	**0.4**
pig 14	2	0.8	0.8	**0.8**	0.4	0.4	**0.4**
pig 14	2.5	0.8	0.8	**0.8**	0.4	0.4	**0.4**

Measurement of myocardial and phrenic nerve threshold by LV-bipolar pacing

LV-bipolar		Myocardial threshold (MT)			PN threshold(PNT)		
	Pulse width (ms)	MT-1	MT-2	mean	PNT-1	PNT-2	mean
pig 1	0.1	4.5	4.6	**4.55**	0.8	0.9	**0.85**
pig 1	0.2	2.4	2.5	**2.45**	0.8	0.8	**0.8**
pig 1	0.3	1.8	1.8	**1.8**	0.8	0.8	**0.8**
pig 1	0.4	1.3	1.4	**1.35**	0.8	0.8	**0.8**
pig 1	0.5	1	1	**1**	0.8	0.8	**0.8**
pig 1	0.7	0.8	0.8	**0.8**	0.8	0.8	**0.8**
pig 1	1	0.6	0.6	**0.6**	0.8	0.8	**0.8**
pig 1	1.5	0.5	0.5	**0.5**	0.8	0.8	**0.8**
pig 1	2	0.4	0.4	**0.4**	0.8	0.8	**0.8**
pig 1	2.5	0.4	0.4	**0.4**	0.8	0.8	**0.8**
pig 2	0.1	6.2	6.4	**6.3**	1.2	1.3	**1.25**
pig 2	0.2	3.3	3.4	**3.35**	1.1	1.1	**1.1**
pig 2	0.3	2.2	2.1	**2.15**	1	1	**1**
pig 2	0.4	1.8	1.8	**1.8**	1	1	**1**
pig 2	0.5	1.5	1.5	**1.5**	0.9	0.9	**0.9**
pig 2	0.7	1.2	1.2	**1.2**	0.8	0.8	**0.8**
pig 2	1	1	1	**1**	0.9	0.9	**0.9**
pig 2	1.5	0.9	0.9	**0.9**	0.9	0.9	**0.9**
pig 2	2	0.8	0.8	**0.8**	0.9	0.9	**0.9**
pig 2	2.5	0.8	0.8	**0.8**	0.8	0.8	**0.8**
pig 3	0.1	9	9.2	**9.1**	1.1	1	**1.05**
pig 3	0.2	5.6	5.8	**5.7**	0.8	0.8	**0.8**
pig 3	0.3	4	4	**4**	0.8	0.8	**0.8**
pig 3	0.4	3.2	3.3	**3.25**	0.8	0.8	**0.8**

pig 3	0.5	2.5	2.5	**2.5**	0.8	0.8	**0.8**
pig 3	0.7	2.1	2.2	**2.15**	0.7	0.7	**0.7**
pig 3	1	1.6	1.6	**1.6**	0.7	0.7	**0.7**
pig 3	1.5	1.6	1.6	**1.6**	0.7	0.7	**0.7**
pig 3	2	1.3	1.3	**1.3**	0.7	0.7	**0.7**
pig 3	2.5	1.3	1.3	**1.3**	0.7	0.7	**0.7**
pig 4	0.1	5.3	5.4	**5.35**	0.8	0.9	**0.85**
pig 4	0.2	4.5	4.6	**4.55**	0.8	0.8	**0.8**
pig 4	0.3	2.8	2.7	**2.75**	0.7	0.7	**0.7**
pig 4	0.4	1	1	**1**	0.7	0.7	**0.7**
pig 4	0.5	0.9	0.9	**0.9**	0.7	0.7	**0.7**
pig 4	0.7	0.7	0.7	**0.7**	0.6	0.6	**0.6**
pig 4	1	0.4	0.4	**0.4**	0.7	0.7	**0.7**
pig 4	1.5	0.2	0.2	**0.2**	0.6	0.6	**0.6**
pig 4	2	0.2	0.2	**0.2**	0.6	0.6	**0.6**
pig 4	2.5	0.2	0.2	**0.2**	0.6	0.6	**0.6**
pig 5	0.1	4.8	4.7	**4.75**	0.8	0.8	**0.8**
pig 5	0.2	3.5	3.4	**3.45**	0.8	0.8	**0.8**
pig 5	0.3	2	2	**2**	0.7	0.8	**0.75**
pig 5	0.4	1.5	1.6	**1.55**	0.7	0.7	**0.7**
pig 5	0.5	1.5	1.5	**1.5**	0.7	0.7	**0.7**
pig 5	0.7	1.4	1.4	**1.4**	0.7	0.7	**0.7**
pig 5	1	1.3	1.3	**1.3**	0.7	0.7	**0.7**
pig 5	1.5	1	1	**1**	0.7	0.7	**0.7**
pig 5	2	0.9	0.9	**0.9**	0.7	0.7	**0.7**
pig 5	2.5	0.7	0.7	**0.7**	0.7	0.7	**0.7**
pig 6	0.1	7.1	7.2	**7.15**	0.4	0.4	**0.4**
pig 6	0.2	3.6	3.7	**3.65**	0.4	0.4	**0.4**
pig 6	0.3	2.7	2.7	**2.7**	0.4	0.4	**0.4**
pig 6	0.4	2.4	2.4	**2.4**	0.4	0.4	**0.4**
pig 6	0.5	1.8	1.8	**1.8**	0.4	0.4	**0.4**
pig 6	0.7	1.5	1.5	**1.5**	0.4	0.4	**0.4**
pig 6	1	1.3	1.3	**1.3**	0.4	0.4	**0.4**
pig 6	1.5	1.1	1.1	**1.1**	0.4	0.4	**0.4**
pig 6	2	0.9	0.9	**0.9**	0.4	0.4	**0.4**

pig 6	2.5	0.8	0.8	**0.8**	0.3	0.3	**0.3**
pig 7	0.1	7.8	7.9	**7.85**	1.1	1.2	**1.15**
pig 7	0.2	5.2	5.3	**5.25**	1.1	1.2	**1.15**
pig 7	0.3	3.9	3.9	**3.9**	1.1	1.1	**1.1**
pig 7	0.4	2.9	3	**2.95**	1	1	**1**
pig 7	0.5	2.4	2.5	**2.45**	1	1	**1**
pig 7	0.7	2	2	**2**	1	1	**1**
pig 7	1	1.6	1.6	**1.6**	1	1	**1**
pig 7	1.5	1.1	1.1	**1.1**	1	1	**1**
pig 7	2	1.1	1.1	**1.1**	1	1	**1**
pig 7	2.5	1	1	**1**	0.9	0.9	**0.9**
pig 8	0.1	3.6	4	**3.8**	0.4	0.5	**0.45**
pig 8	0.2	2	2.1	**2.05**	0.4	0.4	**0.4**
pig 8	0.3	1.8	1.7	**1.75**	0.4	0.4	**0.4**
pig 8	0.4	1.6	1.6	**1.6**	0.4	0.4	**0.4**
pig 8	0.5	1.3	1.4	**1.35**	0.4	0.4	**0.4**
pig 8	0.7	0.8	0.9	**0.85**	0.4	0.4	**0.4**
pig 8	1	0.8	0.8	**0.8**	0.4	0.4	**0.4**
pig 8	1.5	0.7	0.7	**0.7**	0.4	0.4	**0.4**
pig 8	2	0.7	0.7	**0.7**	0.4	0.4	**0.4**
pig 8	2.5	0.7	0.7	**0.7**	0.4	0.4	**0.4**
pig 9	0.1	2.5	2.6	**2.55**	1.8	1.9	**1.85**
pig 9	0.2	1.5	1.6	**1.55**	1.4	1.4	**1.4**
pig 9	0.3	1.1	1.1	**1.1**	1.2	1.2	**1.2**
pig 9	0.4	0.9	0.9	**0.9**	1.1	1.1	**1.1**
pig 9	0.5	0.7	0.7	**0.7**	1.1	1.1	**1.1**
pig 9	0.7	0.6	0.6	**0.6**	1	1	**1**
pig 9	1	0.5	0.5	**0.5**	1	1	**1**
pig 9	1.5	0.4	0.4	**0.4**	0.8	0.8	**0.8**
pig 9	2	0.4	0.4	**0.4**	0.8	0.8	**0.8**
pig 9	2.5	0.4	0.4	**0.4**	0.7	0.7	**0.7**
pig 10	0.1	3.4	3.5	**3.45**	5.6	5.7	**5.65**
pig 10	0.2	2.8	2.6	**2.7**	4.6	4.6	**4.6**
pig 10	0.3	2.1	2	**2.05**	4	4	**4**

pig 10	0.4	2	2	**2**	3.5	3.5	**3.5**
pig 10	0.5	2	1.8	**1.9**	3.5	3.5	**3.5**
pig 10	0.7	2	2.1	**2.05**	3.2	3.2	**3.2**
pig 10	1	1.7	1.8	**1.75**	3	3	**3**
pig 10	1.5	1.6	1.6	**1.6**	2.9	2.9	**2.9**
pig 10	2	1.6	1.6	**1.6**	2.7	2.7	**2.7**
pig 10	2.5	1.6	1.5	**1.55**	2.5	2.5	**2.5**
pig 11	0.1	10	9.8	**9.9**	1.7	1.8	**1.75**
pig 11	0.2	7.7	7.9	**7.8**	1.7	1.7	**1.7**
pig 11	0.3	5.7	5.6	**5.65**	1.7	1.7	**1.7**
pig 11	0.4	5.1	5.1	**5.1**	1.6	1.6	**1.6**
pig 11	0.5	4.6	4.5	**4.55**	1.5	1.5	**1.5**
pig 11	0.7	3.5	3.5	**3.5**	1.7	1.7	**1.7**
pig 11	1	3.2	3.2	**3.2**	1.7	1.7	**1.7**
pig 11	1.5	2.4	2.4	**2.4**	1.7	1.7	**1.7**
pig 11	2	2.4	2.4	**2.4**	1.7	1.7	**1.7**
pig 11	2.5	2	2	**2**	1.7	1.7	**1.7**
pig 12	0.1	3.4	3.5	**3.45**	1.5	1.4	**1.45**
pig 12	0.2	1.9	2	**1.95**	1.4	1.3	**1.35**
pig 12	0.3	1.4	1.4	**1.4**	1.1	1.1	**1.1**
pig 12	0.4	1.3	1.3	**1.3**	0.8	0.8	**0.8**
pig 12	0.5	1.1	1.1	**1.1**	0.8	0.8	**0.8**
pig 12	0.7	1	1	**1**	0.8	0.8	**0.8**
pig 12	1	0.9	0.9	**0.9**	0.8	0.8	**0.8**
pig 12	1.5	0.8	0.8	**0.8**	0.8	0.8	**0.8**
pig 12	2	0.8	0.8	**0.8**	0.8	0.8	**0.8**
pig 12	2.5	0.7	0.7	**0.7**	0.8	0.8	**0.8**
pig 13	0.1	3.8	3.6	**3.7**	1.1	1.2	**1.15**
pig 13	0.2	1.9	2	**1.95**	1	1	**1**
pig 13	0.3	1.2	1.3	**1.25**	1	1	**1**
pig 13	0.4	1.1	1.1	**1.1**	1	1	**1**
pig 13	0.5	0.9	0.9	**0.9**	1	1	**1**
pig 13	0.7	0.7	0.7	**0.7**	1	1	**1**
pig 13	1	0.7	0.7	**0.7**	1	1	**1**
pig 13	1.5	0.6	0.6	**0.6**	1	1	**1**

pig 13	2	0.6	0.6	**0.6**	1	1	**1**
pig 13	2.5	0.6	0.6	**0.6**	1	1	**1**
pig 14	0.1	4	4	**4**	0.5	0.6	**0.55**
pig 14	0.2	3.2	3.1	**3.15**	0.4	0.4	**0.4**
pig 14	0.3	2.6	2.5	**2.55**	0.4	0.4	**0.4**
pig 14	0.4	1.9	1.9	**1.9**	0.4	0.4	**0.4**
pig 14	0.5	1.7	1.7	**1.7**	0.4	0.4	**0.4**
pig 14	0.7	1.3	1.3	**1.3**	0.4	0.4	**0.4**
pig 14	1	1.1	1.1	**1.1**	0.4	0.4	**0.4**
pig 14	1.5	0.9	0.9	**0.9**	0.4	0.4	**0.4**
pig 14	2	0.9	0.9	**0.9**	0.4	0.4	**0.4**
pig 14	2.5	0.9	0.9	**0.9**	0.4	0.4	**0.4**

<u>Effects of Patches on the PN threshold in the LV-tip to RV- coil</u>

	LV-tip ro RV-coil	PN threshold			
	pulse width (ms)	No cover	10*10 mm patch	20*20 mm patch	30*30 mm patch
pig 1	0.5	0.4	2.4	4.6	—
pig 2	0.5	0.5	1	6.1	—
pig 3	0.5	0.4	3.5	4.6	—
pig 4	0.5	0.3	1.2	4.1	—
pig 5	0.5	0.5	2.6	3.8	—
pig 6	0.5	0.2	2.1	4.5	—
pig 7	0.5	0.5	2.2	4.2	—
pig 1	1	0.4	2.1	4.4	—
pig 2	1	0.5	0.9	4.9	—
pig 3	1	0.4	3.1	4.7	—
pig 4	1	0.3	1.5	3.8	—
pig 5	1	0.5	2.8	5.1	—
pig 6	1	0.2	2.7	4.6	—
pig 7	1	0.5	2.9	4.8	—
pig 1	1.5	0.4	2.3	4.5	—
pig 2	1.5	0.5	0.8	4.6	—
pig 3	1.5	0.4	3	3.7	—
pig 4	1.5	0.3	1.9	4.8	—
pig 5	1.5	0.5	2.6	3.8	—

pig 6	1.5	0.2	2.4	3.6	—
pig 7	1.5	0.5	2.7	3.1	—
pig 1	2	0.4	2.2	4.8	—
pig 2	2	0.5	0.8	4.4	—
pig 3	2	0.4	3	3.4	—
pig 4	2	0.3	2.1	3.8	—
pig 5	2	0.5	2.6	3.7	—
pig 6	2	0.2	1.9	4.1	—
pig 7	2	0.5	1.8	4.3	—
pig 1	2.5	0.4	2.1	4.7	—
pig 2	2.5	0.5	0.8	4	—
pig 3	2.5	0.4	2.8	3.2	—
pig 4	2.5	0.3	1.8	3.3	—
pig 5	2.5	0.5	2.3	3.6	—
pig 6	2.5	0.2	1.8	3.9	—
pig 7	2.5	0.5	1.6	3.7	—

—: no phrenic stimulation

Effects of Patches on the PN threshold in the LV-tip to RV- ring

	LV-tip ro RV-ring	PN threshold			
	pulse width (ms)	No cover	10*10 mm patch	20*20 mm patch	30*30 mm patch
pig 1	0.5	0.5	3.2	3.3	—
pig 2	0.5	0.8	1.3	7.4	—
pig 3	0.5	0.5	4.1	4.5	—
pig 4	0.5	1.1	2.5	4.7	—
pig 5	0.5	0.7	4.4	5.2	—
pig 6	0.5	0.3	4.1	4.8	—
pig 7	0.5	0.8	3.3	4.2	—
pig 1	1	0.5	3	3.5	—
pig 2	1	0.7	1.1	6.8	—
pig 3	1	0.5	3.8	4.1	—
pig 4	1	1	2.1	4.5	—
pig 5	1	0.7	4.3	5	—
pig 6	1	0.3	4	4.5	—
pig 7	1	0.8	3.2	4	—

pig 1	1.5	0.5	2.9	3.4	—
pig 2	1.5	0.7	1.1	6.4	—
pig 3	1.5	0.5	3.7	4	—
pig 4	1.5	1	1.9	4.1	—
pig 5	1.5	0.7	4.3	4.7	—
pig 6	1.5	0.3	3.9	4.4	—
pig 7	1.5	0.8	3.3	3.7	—
pig 1	2	0.5	3.1	3.3	—
pig 2	2	0.8	1.2	5.7	—
pig 3	2	0.5	3.6	4.1	—
pig 4	2	1	1.8	3.8	—
pig 5	2	0.7	4.3	4.5	—
pig 6	2	0.3	3.6	4.1	—
pig 7	2	0.8	3.1	3.6	—
pig 1	2.5	0.5	3.2	3	—
pig 2	2.5	0.7	1.1	5.6	—
pig 3	2.5	0.5	3.6	3.9	—
pig 4	2.5	1	1.8	3.8	—
pig 5	2.5	0.7	4.1	4.4	—
pig 6	2.5	0.3	3.2	3.8	—
pig 7	2.5	0.8	3	3.5	—

—: no phrenic stimulation

Effects of Patches on the PN threshold in the LV-bipolar

	LV-bipolar pulse width (ms)	PN threshold		
		No cover	10*10 mm patch	20*20 mm patch
pig 1	0.5	0.8	9.9	—
pig 2	0.5	0.9	4.3	—
pig 3	0.5	0.8	8.4	—
pig 4	0.5	0.7	6	—
pig 5	0.5	0.7	7	—
pig 6	0.5	0.4	7.3	—
pig 7	0.5	1	6.8	—

pig 1	1	0.8	8.9	—
pig 2	1	0.9	3.8	—
pig 3	1	0.7	8.3	—
pig 4	1	0.7	5.5	—
pig 5	1	0.7	6.8	—
pig 6	1	0.4	7.1	—
pig 7	1	1	6.8	—
pig 1	1.5	0.8	8.1	—
pig 2	1.5	0.9	3.7	—
pig 3	1.5	0.7	8.4	—
pig 4	1.5	0.6	5.3	—
pig 5	1.5	0.7	6.6	—
pig 6	1.5	0.4	6.9	—
pig 7	1.5	1	6.5	—
pig 1	2	0.8	8	—
pig 2	2	0.9	3.8	—
pig 3	2	0.7	7.7	—
pig 4	2	0.6	5.2	—
pig 5	2	0.7	6.3	—
pig 6	2	0.4	6.5	—
pig 7	2	1	6.1	—
pig 1	2.5	0.8	7.9	—
pig 2	2.5	0.8	3.8	—
pig 3	2.5	0.7	7.6	—
pig 4	2.5	0.6	5	—
pig 5	2.5	0.7	6.2	—
pig 6	2.5	0.3	6.4	—
pig 7	2.5	0.9	6	—

—: no phrenic stimulation

Effects of Graft on the PN threshold in the LV-tip to RV-coil

	LV-tip ro RV-coil	PN threshold			
	pulse width (ms)	No cover	10 mm graft	20 mm graft	30 mm graft
pig 8	0.5	0.3	1.7	3.5	—
pig 9	0.5	0.5	1.7	6.2	—
pig 10	0.5	1.5	2.3	6.4	—
pig 11	0.5	0.9	1.6	3.6	—
pig 12	0.5	0.4	1.5	4.5	—
pig 13	0.5	0.5	2.4	4.5	—
pig 14	0.5	0.3	1.2	2.4	—
pig 8	1	0.3	1.6	3.4	—
pig 9	1	0.5	1.5	5.6	—
pig 10	1	1.6	2.1	6	—
pig 11	1	0.8	1.4	3.3	—
pig 12	1	0.4	1.4	4	—
pig 13	1	0.5	2.1	3.8	—
pig 14	1	0.3	1.2	2.4	—
pig 8	1.5	0.3			—
pig 9	1.5	0.5	1.5	5.5	—
pig 10	1.5	1.7	2.1	5.9	—
pig 11	1.5	0.7	1.3	3.3	—
pig 12	1.5	0.4	1.4	3.3	—
pig 13	1.5	0.5	2.1	3.1	—
pig 14	1.5	0.3	1.2	2.4	—
pig 8	2	0.2	1.7	3.4	—
pig 9	2	0.5	1.4	5.2	—
pig 10	2	1.7	2.1	5.9	—
pig 11	2	0.7	1.2	3.2	—
pig 12	2	0.4	1.4	3.3	—
pig 13	2	0.5	2	3	—
pig 14	2	0.3	1.1	2.4	—
pig 8	2.5	0.2	1.6	3.4	—
pig 9	2.5	0.5	1.4	5.2	—
pig 10	2.5	1.6	2.1	5.8	—

pig 11	2.5	0.6	1.1	3.2	—
pig 12	2.5	0.4	1.4	3.3	—
pig 13	2.5	0.5	2	2.8	—
pig 14	2.5	0.3	1.1	2.3	—

Effects of Graft on the PN threshold in the LV-tip to RV-ring

	LV-tip ro RV-ring	PN threshold			
	pulse width (ms)	No cover	10 mm graft	20 mm graft	30 mm graft
pig 8	0.5	0.4	2.9	4	—
pig 9	0.5	0.8	3.4	10	—
pig 10	0.5	2.5	3.3	8	—
pig 11	0.5	1.7	3.4	6.4	—
pig 12	0.5	0.4	2	6.5	—
pig 13	0.5	0.9	4.6	7	—
pig 14	0.5	0.4	2	3.5	—
pig 8	1	0.4	2.8	3.8	—
pig 9	1	0.7	3.4	10	—
pig 10	1	2.6	3.1	7.3	—
pig 11	1	1.5	3	5.5	—
pig 12	1	0.4	1.8	5.2	—
pig 13	1	0.9	4.1	6.1	—
pig 14	1	0.4	2	3.3	—
pig 8	1.5	0.4	2.5	3.6	—
pig 9	1.5	0.7	3.4	9.8	—
pig 10	1.5	2.6	3	6.9	—
pig 11	1.5	1.6	2.9	5.5	—
pig 12	1.5	0.4	1.7	5.1	—
pig 13	1.5	0.9	4	6	—
pig 14	1.5	0.4	1.9	3.3	—
pig 8	2	0.4	2.5	3.6	—
pig 9	2	0.7	3.3	9.7	—
pig 10	2	2.6	3	6.9	—
pig 11	2	1.6	2.8	5.5	—
pig 12	2	0.4	1.7	4.8	—

pig 13	2	0.9	3.9	6	—
pig 14	2	0.4	1.9	3.3	—
pig 8	2.5	0.4	2.4	3.6	—
pig 9	2.5	0.7	2.4	3.6	—
pig 10	2.5	2.4	3.3	9.5	—
pig 11	2.5	1.5	3	6.9	—
pig 12	2.5	0.4	2.8	5.3	—
pig 13	2.5	0.9	1.6	4.5	—
pig 14	2.5	0.4	3.7	6	—

Effects of Graft on the PN threshold in the LV-bipolar

	LV-bipolar	PN threshold			
	pulse width (ms)	No cover	10 mm graft	20 mm graft	Post procedure
pig 8	0.5	0.4	5.8	—	0.5
pig 9	0.5	1.1	8.3	—	1.1
pig 10	0.5	3.8	10	—	3.6
pig 11	0.5	1.5	3.4	—	1.4
pig 12	0.5	0.8	3.2	—	0.9
pig 13	0.5	1	10	—	0.9
pig 14	0.5	0.4	3.5	—	0.5
pig 8	1	0.4	5.6	—	0.5
pig 9	1	1	7.4	—	0.9
pig 10	1	3	10	—	3.1
pig 11	1	1.7	2.9	—	1.5
pig 12	1	0.8	2.9	—	0.9
pig 13	1	1	10	—	0.9
pig 14	1	0.4	3.4	—	0.5
pig 8	1.5	0.4	5.6	—	0.5
pig 9	1.5	0.8	6.9	—	0.9
pig 10	1.5	2.9	10	—	2.8
pig 11	1.5	1.7	2.2	—	1.8
pig 12	1.5	0.8	2.9	—	1
pig 13	1.5	1	10	—	0.7
pig 14	1.5	0.4	3.3	—	0.5

pig 8	2	0.4	5.6	—	0.5
pig 9	2	0.8	6.9	—	0.8
pig 10	2	2.7	10	—	3
pig 11	2	1.7	1.8	—	1.8
pig 12	2	0.8	2.8	—	0.9
pig 13	2	1	10	—	0.9
pig 14	2	0.4	3.3	—	0.5
		0.4			
pig 8	2.5	0.7	5.5	—	0.5
pig 9	2.5	2.5	6.9	—	0.8
pig 10	2.5	1.7	10	—	3
pig 11	2.5	0.8	1.5	—	1.8
pig 12	2.5	1	2.8	—	0.6
pig 13	2.5	0.4	10	—	0.9
pig 14	2.5	0.4	3.3	—	0.5
